# Femoral neck anteversion- what’s normal in Indians: a morphological study of 742 hips using CT-based MAKO robotic evaluation

**DOI:** 10.1186/s12891-026-10041-6

**Published:** 2026-06-09

**Authors:** Adarsh Annapareddy, Tarun Jayakumar, Sneh Shah, Mahesh Madhukar Kulkarni, Arun Kannan, Ravikumar Mukartihal, Sharan S. Patil, Rohit Rakesh Luthra, Ashish Singh, Vemaganti Badri Narayana Prasad, Mujtaba Ansari, A. V. Gurava Reddy

**Affiliations:** 1 Department of Orthopaedics, Sunshine Bone and Joint Institute, KIMS-Sunshine Hospitals, Hyderabad, India; 2 Department of Orthopaedics, Deenanath Mangeshkar Hospital, Pune, India; 3 Department of Orthopaedics, Apollo Hospitals, Greams Road, Chennai, India; 4 Department of Orthopaedics, Sparsh Hospital, Bangalore, India; 5 Department of Orthopaedics, Arcus Hospital, Pune, India; 6 Department of Orthopaedics, Anup Institute of Orthopaedics and Rehabilitation, Patna, India

**Keywords:** Femoral neck anteversion, Femoral version, Femoral retroversion, Total hip arthroplasty, Robotic-assisted total hip arthroplasty, CT-based planning, MAKO robotic system, Indian population, Hip morphology, Combined anteversion

## Abstract

**Background:**

Femoral neck anteversion (FNA) is a key determinant of hip biomechanics and component positioning in total hip arthroplasty (THA). Most reference values for femoral version are derived from Western populations and non-robotic measurement techniques. Data defining native femoral version in the Indian population using CT-based robotic planning are limited.

**Methods:**

This multicenter cross-sectional study analyzed 742 hips undergoing primary robotic-assisted THA using the MAKO THA 4.0 system. Native femoral version was calculated automatically from preoperative CT scans as the angle between the femoral neck axis and the trans-epicondylar axis. Femoral version was categorized as retroversion (< 0°), neutral (0°), or anteversion (> 0°). Descriptive statistics were reported as Mean (SD). Group comparisons were performed using chi-square tests, with statistical significance set at *p* < 0.05.

**Results:**

The mean native femoral version was − 2.6° (SD 11.9), with a range from − 42° to 41°. Overall, 395 hips (53.2%) demonstrated retroversion, 320 hips (43.1%) anteversion, and 27 hips (3.6%) neutral alignment. Retroversion predominated in males, whereas females showed significantly higher anteversion (*p* < 0.001). No statistically significant differences were observed across age groups (*p* = 0.310; Spearman ρ = 0.034, *p* = 0.356) or diagnosis categories (*p* = 0.227). Notably, 703 hips (94.7%) demonstrated femoral version below 15°.

**Conclusion:**

Indian patients undergoing THA demonstrate substantially lower femoral neck anteversion than commonly cited Western norms, with more than half exhibiting femoral retroversion. These findings highlight the importance of population-specific and individualized preoperative planning, for which CT-based assessment can be useful.

## Introduction

Femoral neck anteversion represents the axial angular relationship between the femoral neck and distal femoral condyles and plays a fundamental role in hip biomechanics, range of motion, and stability following total hip arthroplasty (THA) [1]. Aberrant femoral version has been associated with impingement, instability, altered contact stresses, and abnormal wear patterns [2–4]. Traditionally, “normal” femoral anteversion has been described as ranging between 10° and 15°; however, published values vary widely depending on population characteristics, measurement techniques, and reference axes [5–7]. Ethnic and lifestyle-related differences in femoral morphology have long been postulated. Anthropometric and radiological studies from India and other Asian populations have consistently reported lower mean anteversion compared with Western cohorts [2, 8–11]. Cultural practices such as squatting and floor sitting, as well as developmental factors, have been suggested as possible contributors [11, 12]. Despite this, most existing Indian data are derived from cadaveric specimens, plain radiography, or conventional CT, all of which are subject to substantial interobserver and methodological variability [13–15]. 

The advent of robotic-assisted THA has enabled standardized, CT-based preoperative planning with improved reproducibility of anatomical measurements [16, 17]. Robotic systems allow automated calculation of femoral version using consistent anatomic landmarks, minimizing observer bias and enabling large-scale, multicenter evaluation of native anatomy [18, 19]. Given the lack of large-scale standardized CT-based data in Indian populations, and prior reports suggesting lower femoral anteversion in Asian cohorts, we anticipated that native femoral version in Indian patients may differ from traditionally reported Western values.

The primary objective of this study was to define the distribution of native femoral neck anteversion in an Indian population undergoing robotic-assisted THA using MAKO 4.0 software. Secondary objectives included evaluating variation by age, sex, and underlying etiology.

## Methods

### Study design and participant selection

This was a multicenter retrospective cross-sectional study conducted across 6 high-volume tertiary arthroplasty centers in India and adhered to the STROBE guidelines for observational studies [20]. Institutional ethics committee approval was obtained (SIEC/2023/530) prior to the start of the study and anonymized patient data was used from all participating centers.

### Patient selection

A total of 846 consecutive hips undergoing primary robotic-assisted THA between July 2022 and June 2023 were initially screened for inclusion. Hips with developmental dysplasia of the hip (defined as Crowe grade II or higher), post-traumatic deformity (including malunion or prior fracture involving the proximal femur), prior hip surgery, ankylosing spondylitis with hip ankylosis, neuromuscular disorders affecting femoral torsion, and significant proximal femoral morphological abnormalities were excluded. After exclusions, 742 hips were available for final analysis. The cohort included patients aged 20 to 80 years, with avascular necrosis (AVN) and primary osteoarthritis (OA) as the predominant diagnoses.

### CT imaging and measurement of femoral version

All patients underwent CT imaging which includes thin-slice axial imaging (slice thickness ≤ 1 mm) from the pelvis to the distal femoral condyles, with patients positioned supine and lower limbs maintained in neutral rotation as a part of the MAKO robotic planning protocol (Stryker Corp, Kalamazoo, MI). In addition to standardized CT acquisition protocols, segmentation and preoperative planning for all cases were performed by a centralized MAKO planning team, ensuring uniformity in three-dimensional reconstruction and landmark identification. This centralized processing minimizes inter-center variability and enhances consistency of femoral version measurements across the study cohort. Native femoral neck anteversion was calculated automatically by the MAKO THA 4.0 software [21]. 

**Fig. 1:**
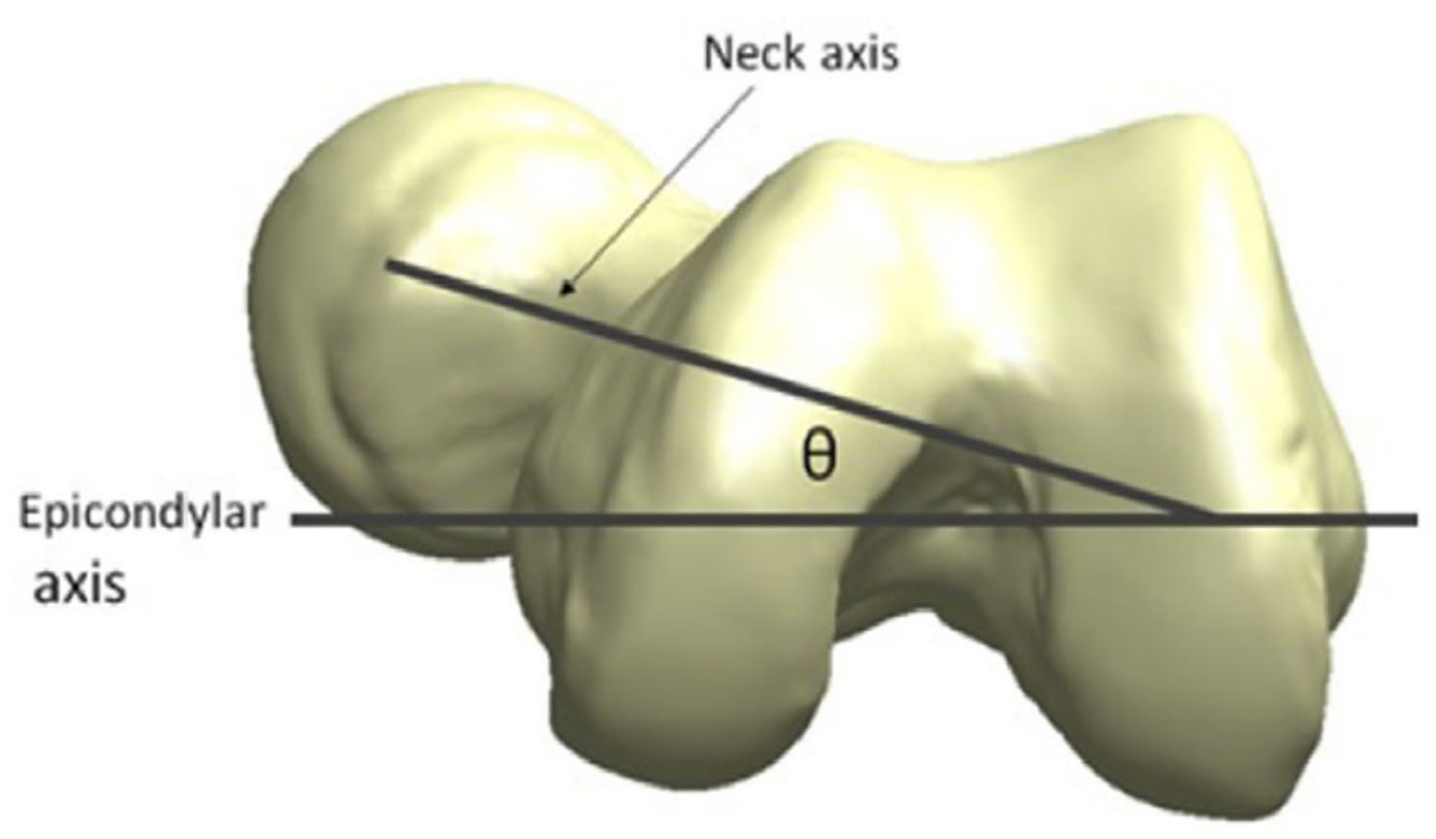
Native femoral neck anteversion is measured as the angle between the femur neck axis and trans-epicondylar axis when these 2 axes are projected on a plane perpendicular to the anatomic axis of the femur

**Fig. 2:**
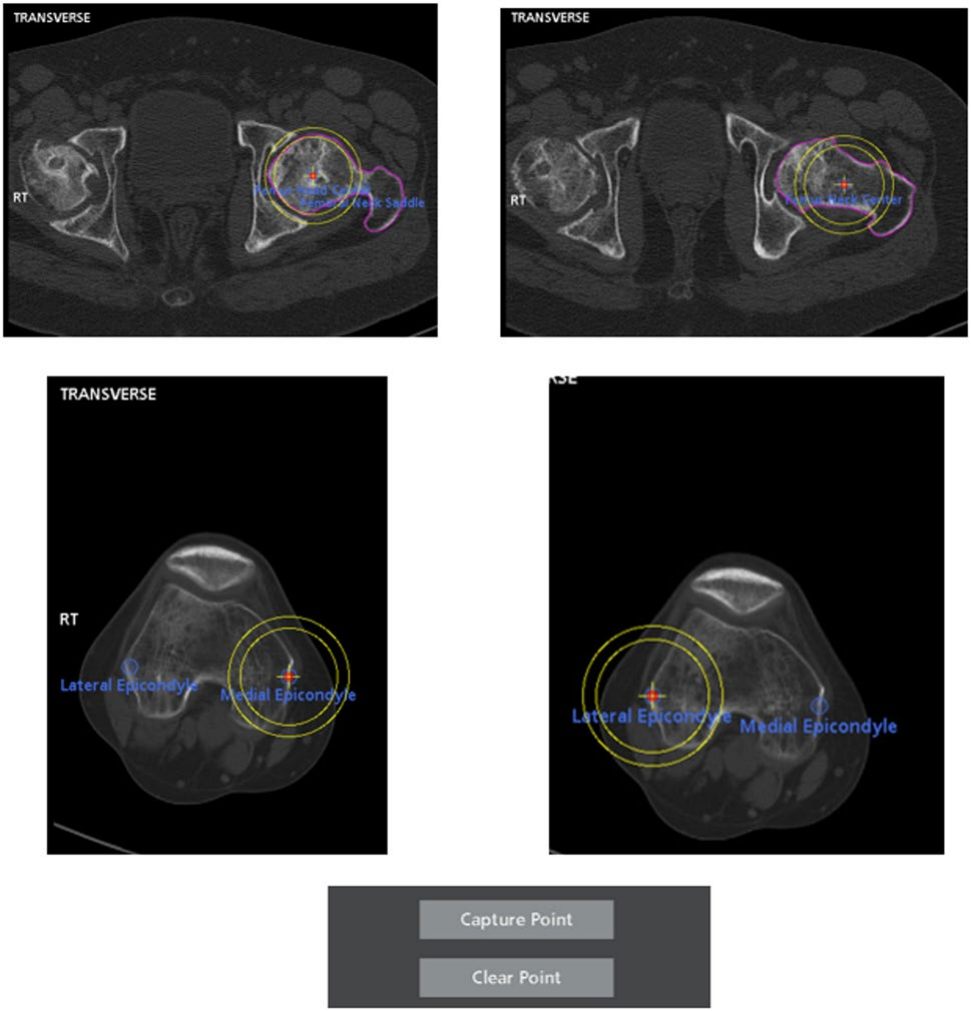
Capturing landmarks on the MAKO robotic screen

The femoral neck axis was defined as the line connecting the center of the femoral head and the center of the femoral neck. The distal femoral reference was established by the surgical transepicondylar axis (TEA), defined as the line joining the sulcus of the medial epicondyle and the prominence (tip) of the lateral epicondyle of the distal femur. (Fig. 1) Anteversion was defined as the angle between these two axes in the axial plane. (Fig. 2) The posterior condylar axis (PCA) has traditionally been used as a reference for femoral anteversion measurement. However, in the present study, femoral version was measured relative to the surgical transepicondylar axis (TEA), as defined by the MAKO robotic planning protocol. The use of standardized robotic CT-based landmarks reduces observer-dependent variability and facilitates large-scale, multicenter evaluation.

### Statistical analysis

Statistical analysis was done using SPSS software version 24.0 (IBM, Armonk, NY, USA). Continuous variables were reported as Mean (SD) and ranges. Normality of distribution was assessed using the Shapiro-Wilk test. As femoral neck anteversion demonstrated a non-normal distribution, continuous data were summarized using both mean with standard deviation (SD) and median with interquartile range (IQR). Ranges were reported where appropriate. 95% confidence intervals (95% CI) were calculated for key continuous estimates to improve the precision and interpretability of the reported values. Additional subgrouping into arbitrary version categories was avoided to reduce the risk of information loss, misclassification bias, and disproportionate subgroup sizes. For descriptive categorical analysis, femoral version was classified as retroversion (< 0°), neutral (= 0°), and anteversion (> 0°).

Categorical variables were summarized as frequencies and percentages. Comparisons of femoral version category distribution across age group, sex, and diagnosis were performed using the chi-square test of independence. For continuous femoral version comparisons, the Mann-Whitney U test was used for two-group comparisons, and the Kruskal-Wallis test was used for comparisons involving more than two groups. The association between age and femoral version was assessed using Spearman’s rank correlation coefficient. A two-tailed p-value less than 0.05 was considered statistically significant.

As this was a retrospective cross-sectional study including all eligible cases within the study period, a formal a priori sample size calculation was not performed. However, the present cohort represents one of the largest multicenter CT-based datasets evaluating femoral version in an Indian population, exceeding the sample sizes reported in prior literature.

## Results

A total of 742 hips were analyzed following exclusion of hips with dysplasia, ankylosing spondylitis, and post-traumatic arthritis. The mean age of the cohort was 43.4 years (SD = 13.5). There were 580 males (78.0%) and 162 females (21.8%). Avascular necrosis was the predominant indication for THA, accounting for 663 hips (89.3%), followed by primary osteoarthritis in 63 hips (8.5%).

**Fig. 3:**
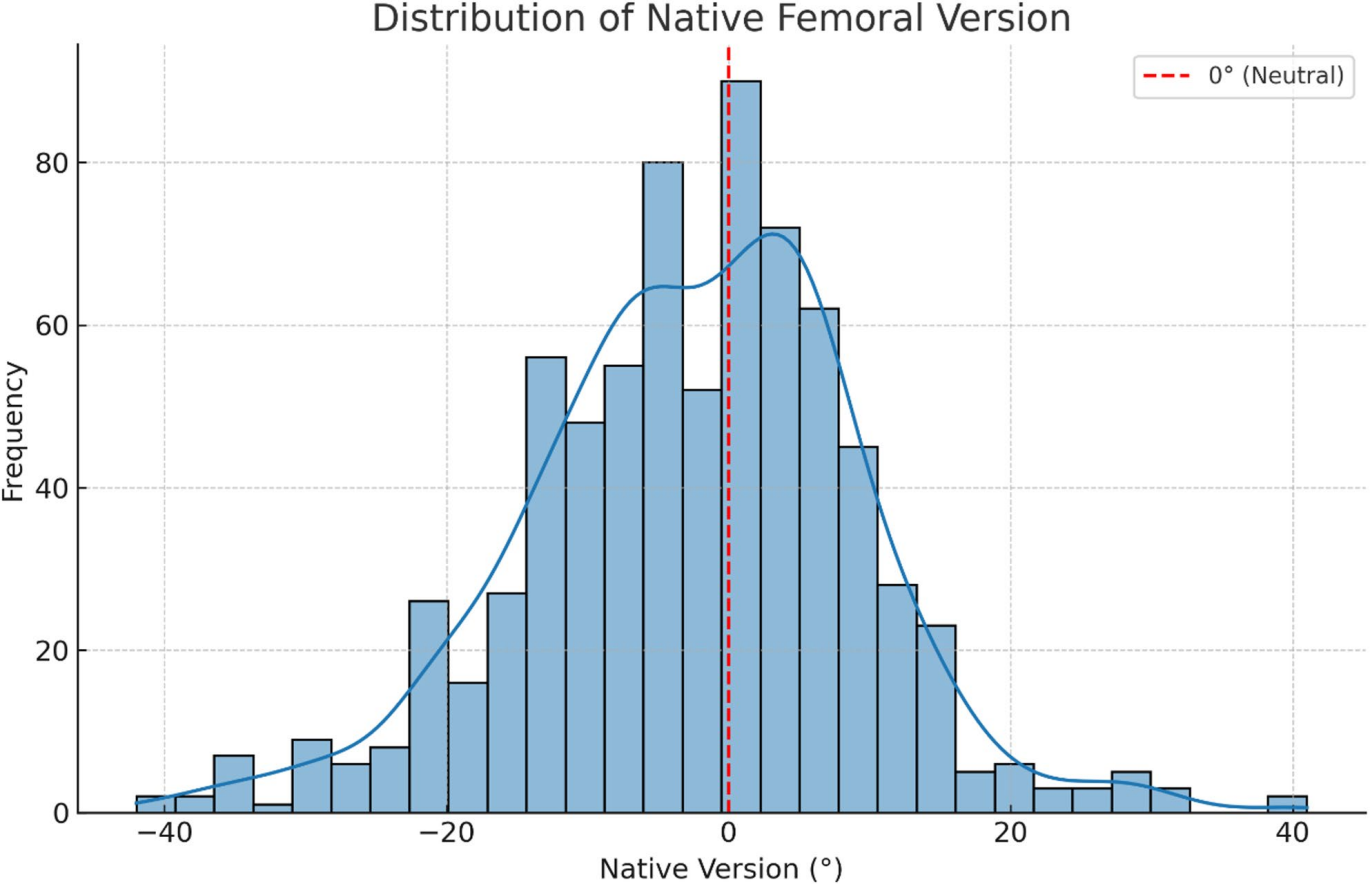
Overall distribution of Femur version in the study population

The overall mean native femoral neck anteversion was − 2.6° (SD 11.9; 95% CI, -3.33 to -1.60), with a median of -3° (IQR, -11 to 6). The values ranged from − 42° to 41°, demonstrating a wide dispersion of femoral version in the study population. (Fig. 3) Overall, 395 hips (53.2%) demonstrated femoral retroversion, 320 hips (43.1%) demonstrated anteversion, and 27 hips (3.6%) were neutral (Table 1).

**Table 1:** Distribution of femoral version categories by age group, gender, and diagnosis

Parameter	Subgroup	Total	Anteversion (degrees) *n* (%)	Neutral (degrees) *n* (%)	Retroversion (degrees) *n* (%)	*p*-value
Age Group (years)	< 40	365	145 (39.7)	16(4.4)	204 (55.9)	*0.382
	40–49	154	68 (44.2)	3 (1.9)	83 (53.9)	
	50–59	114	58 (50.9)	6 (5.2)	50 (43.9)	
	60–69	70	29 (41.4)	2 (2.9)	39 (55.7)	
	70–79	33	16 (48.5)	0	17 (51.5)	
	80+	6	4 (66.7)	0	2 (33.3)	
Gender	Female	162	99 (61.1)	2 (1.2)	61 (37.7)	*<0.001
	Male	580	221 (38.1)	25 (4.3)	334 (57.6)	
Diagnosis	Avascular Necrosis	663	280 (42.2)	25 (3.8)	358 (54)	*0.304
	Osteoarthritis	63	35 (55.6)	2 (3.2)	26 (41.2)	
	Others	16	8 (50)	1 (6.3)	7 (43.7)	

*- Chi-square test

### Age-stratified analysis

**Fig. 4:**
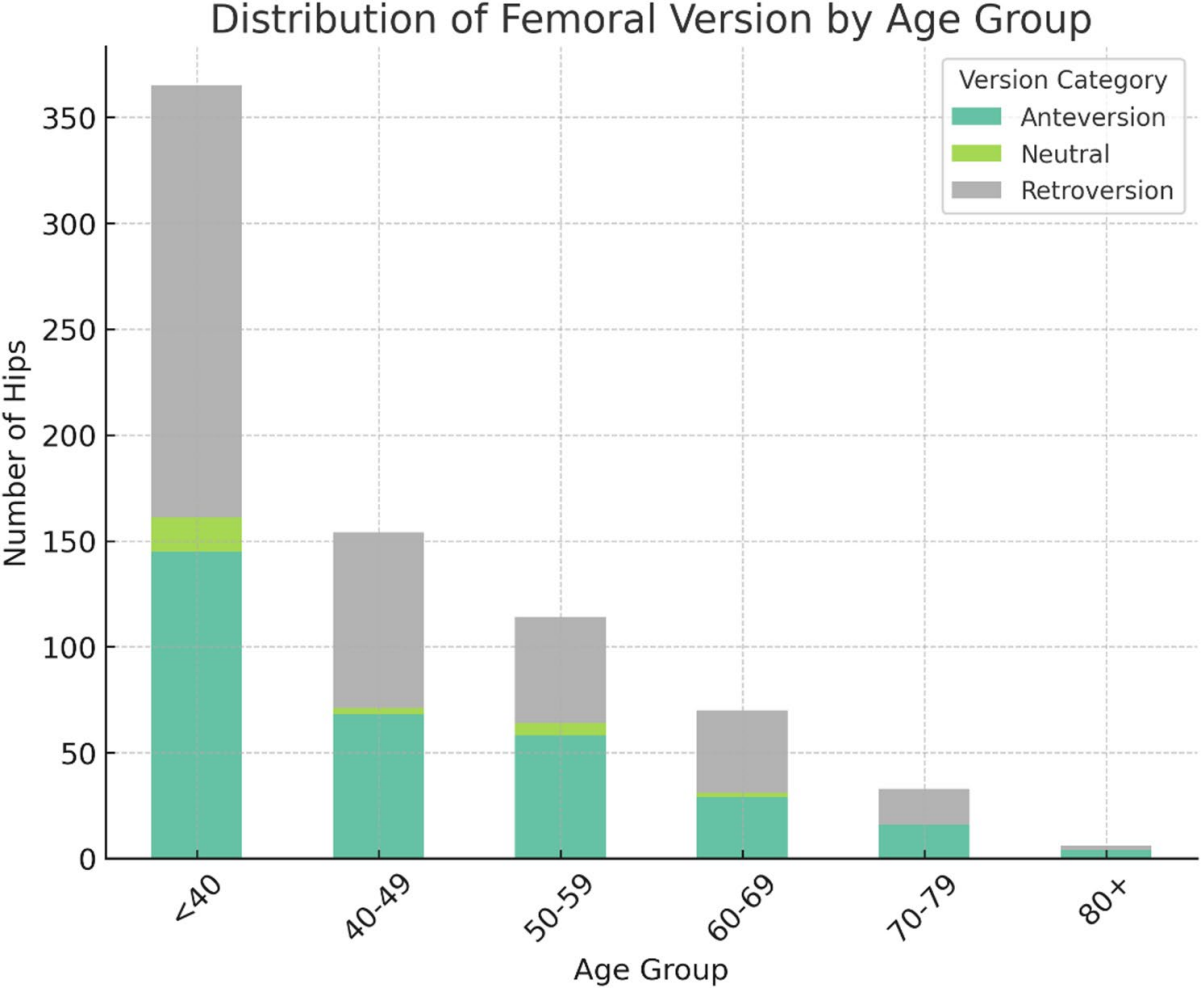
Distribution of femur version based on age-group

Age-stratified analysis demonstrated no statistically significant difference in femoral version distribution across age groups (chi-square, *p* = 0.382). Retroversion was most frequently observed in patients younger than 40 years, but this trend did not reach statistical significance. (Fig. 4)

Continuous analysis similarly demonstrated no significant difference in femoral version across age groups (*p* = 0.310). The mean femoral version was − 3.5° (SD 12.1) in patients younger than 40 years, -2.8° (SD 11.4) in those aged 40–49 years, -1.6° (SD 10.9) in those aged 50–59 years, -3.2° (SD 11.8) in those aged 60–69 years, and − 0.9° (SD 10.7) in patients aged 70 years or older. Median values were also comparable across age groups, ranging from − 4° to -1°. (Table 2)

**Table 2:** Mean and median femoral version measurements across subgroups

Parameter	Subgroup	*n*	FNA (°) Mean (SD)	95% CI (°)	FNA (°) Median (IQR)	*p*-value
Age Group (years)	< 40	365	−3.5 (12.1)	−4.7 to − 2.2	−4 (− 12 to 5)	† 0.310
	40–49	154	−2.8 (11.4)	−4.6 to − 1.0	−3 (− 10 to 6)	
	50–59	114	−1.6 (10.9)	−3.6 to 0.4	−2 (− 9 to 7)	
	60–69	70	−3.2 (11.8)	−6.0 to − 0.4	−3 (− 11 to 5)	
	≥ 70	39	−0.9 (10.7)	−4.4 to 2.6	−1 (− 8 to 8)	
Gender	Male	580	−3.8 (11.7)	−4.8 to − 2.9	−4 (− 11 to 4)	* <0.001
	Female	162	2.1 (12.0)	0.2 to 4.0	2.5 (− 5 to 10)	
Diagnosis	Avascular Necrosis	663	−2.9 (11.8)	−3.8 to − 2.0	−3 (− 11 to 5)	† 0.227
	Osteoarthritis	63	1.8 (11.2)	−1.0 to 4.6	2 (− 6 to 9)	
	Others	16	0.5 (14.2)	−6.3 to 7.4	−1.0 (− 11.5 to 8.0)	

†- Kruskal-Wallis test; * - Mann-Whitney U test

Spearman correlation analysis showed no significant association between age and femoral version (ρ = 0.034, *p* = 0.356), confirming the absence of a meaningful age-related trend (Table 3).

**Table 3:** Association of femoral version with sex and age

Variable	Subgroup	*n*	Mean (SD) FNA (°)	95% CI (°)	Median (IQR) (°)	Range (°)	Statistical Test	*p*-value
Gender	Male	580	-3.8 (11.7)	-4.72 to -2.82	-4 (-11 to 4)	-42 to 41	Mann-Whitney U	< 0.001
	Female	162	2.1 (12.0)	0.26 to 3.94	2.5 (-5 to 10)	-29 to 41		
Age	Overall	742	-2.6 (11.9)	-3.33 to -1.60	-3 (-11 to 6)	-42 to 41	Spearman correlation ρ = 0.034	0.356

### Sex-based analysis

**Fig. 5:**
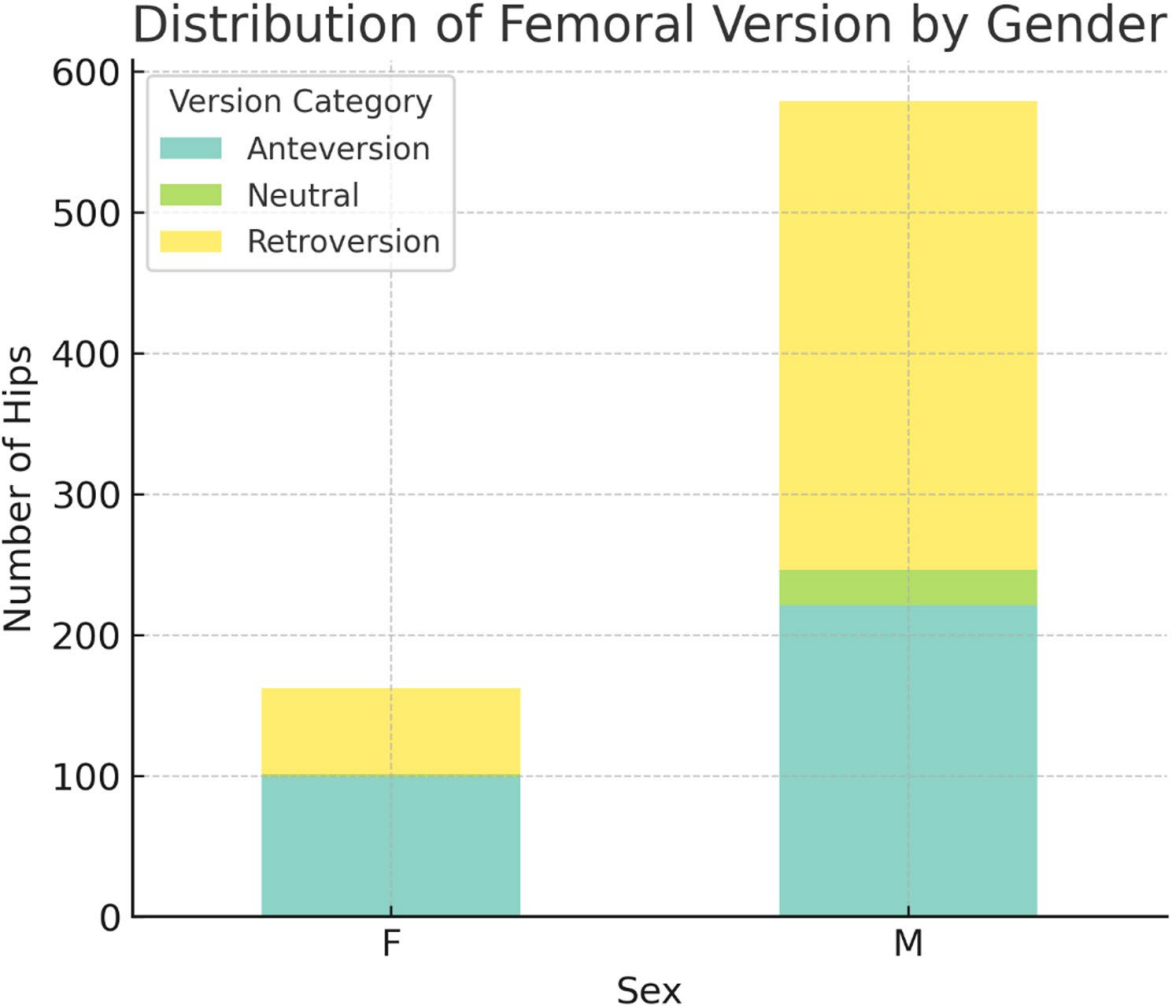
Distribution of femur version based on gender. AVN- Avascular Necrosis; OA- Osteoarthritis

Sex-based analysis demonstrated a significant difference in femoral version distribution (Fig. 5). Among females, 99 hips (61.1%) demonstrated anteversion, 2 hips (1.2%) were neutral, and 61 hips (37.7%) were retroverted. Among males, 221 hips (38.1%) demonstrated anteversion, 25 hips (4.3%) were neutral, and 334 hips (57.6%) were retroverted. (*p* < 0.001) (Table 1).

Continuous femoral version analysis confirmed this sex-based difference. Males demonstrated a mean femoral version of -3.8° (SD 11.7; 95% CI, -4.72 to -2.82), with a median of -4° (IQR, -11 to 4) and a range from − 42° to 41°. Females demonstrated a mean femoral version of 2.1° (SD 12.0; 95% CI, 0.26 to 3.94), with a median of 2.5° (IQR, -5 to 10) and a range from − 29° to 41° (Table 2). This difference was statistically significant on Mann-Whitney U testing (*p* < 0.001), indicating that females had significantly greater femoral anteversion than males (Table 3).

### Diagnosis-based analysis

**Fig. 6:**
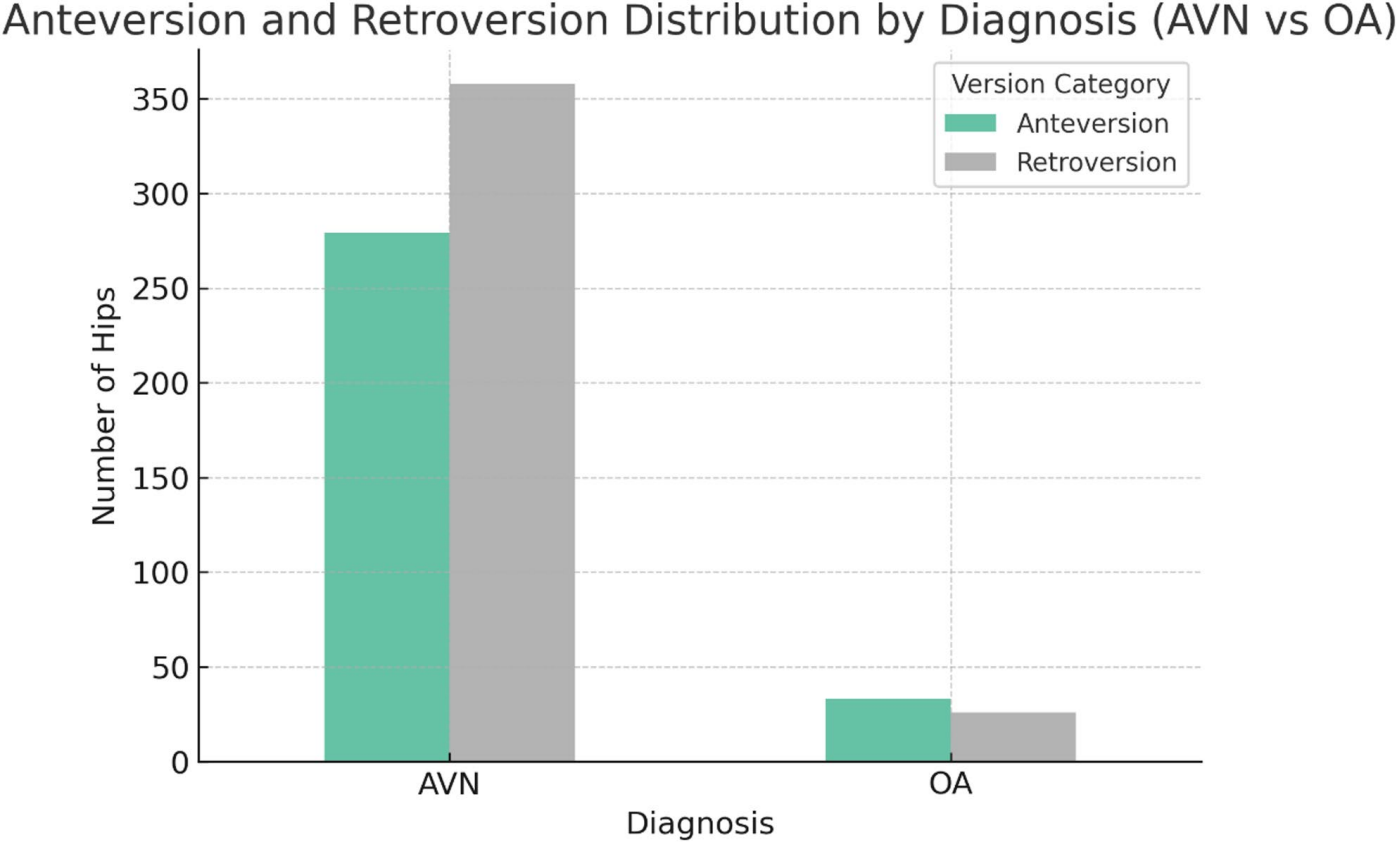
Distribution of femur version based on diagnosis

Comparison based on etiology revealed no statistically significant difference between AVN and primary OA (Fig. 6). The distribution of femoral version categories did not differ significantly across diagnosis groups (chi-square test, *p* = 0.304). Among hips with avascular necrosis, 280 hips (42.2%) were anteverted, 25 hips (3.8%) were neutral, and 358 hips (54.0%) were retroverted. Among hips with osteoarthritis, 35 hips (55.6%) were anteverted, 2 hips (3.2%) were neutral, and 26 hips (41.2%) were retroverted. In the others group, 8 hips (50.0%) were anteverted, 1 hip (6.3%) was neutral, and 7 hips (43.7%) were retroverted (Table 1).

Continuous analysis also demonstrated no statistically significant difference in femoral version across diagnosis groups (*p* = 0.227). Hips with avascular necrosis had a mean femoral version of -2.9° (SD 11.8), with a median of -3° (IQR, -11 to 5). Hips with osteoarthritis had a mean femoral version of 1.8° (SD 11.2), with a median of 2° (IQR, -6 to 9). The others group had a mean femoral version of 0.5° (SD 14.2), with a median of -1.0° (IQR, -11.5 to 8.0). These findings suggest that femoral version was broadly comparable across diagnostic categories (Table 2).

Importantly, 703 of the 742 hips analyzed (94.7%) demonstrated native femoral version less than 15°, indicating that the vast majority of patients fell below the traditionally cited “normal” anteversion threshold.

## Discussion

The most important finding of the present multicenter CT-based robotic study is that native femoral neck anteversion in Indian patients undergoing primary THA is substantially lower than traditionally cited Western norms, with a mean value of -2.6° and more than half of hips demonstrating femoral retroversion. Furthermore, 94.7% of hips demonstrated femoral version < 15°, challenging the long-standing assumption that a “physiological” anteversion range of 10°-15° is universally applicable.

### Comparison with existing literature

The predominance of femoral retroversion observed in this study exceeds that reported in most prior Indian CT-based analyses, which have typically described low but positive mean anteversion values. The higher prevalence of retroversion in the present study is likely attributable to the technique used on standardized robotic CT-based measurement, which minimizes observer variability and employs a consistent condylar reference axis. Earlier studies relying on plain radiographs or non-standardized CT techniques may have underestimated mild degrees of retroversion by classifying such hips as neutral [14, 22]. Patro et al., in an Eastern Indian cohort, highlighted significant discrepancies between clinical, radiographic, and CT-based measurements, with CT yielding lower and more reliable values [12]. However, their study was limited by small sample size and non-standardized landmarks. The present study overcomes these limitations by leveraging automated MAKO 4.0 CT-based calculations using standardized transepicondylar axis (TEA) referencing, providing one of the largest multicenter CT-based analyses of femoral version in an Indian population using standardized robotic measurement (Table 4).

**Table 4:** Native femoral version reported by various authors

Author / Year	Country / Population	Sample	Method	Mean ± SD (°)	Notes
Kingsley & Olmsted, 1948	USA, cadaver femora	1,600	Dry bone	~ 15	Classic Western baseline
Harrison, 1953	UK, cadaver + OA hips	136	Radiographs / pathology	Variable	OA hips altered
Reikerås, 1983	Norway	86	CT	13 ± 7 (normals); 19 (OA)	OA anteversion higher
Murphy, 1987 (JBJS)	USA	215	CT (Murphy method)	28 ± 13	Method-dependent
Srimathi, 2012	India, cadaver femur	164	Goniometer	9.8	Indian values lower
Jain, 2005 (IJO)	India	300 dry, 72 CT, 138 XR, 138 clinical	Multiple	CT 7.4 ± 4.6; Dry 8.1	Radiographs & clinical higher
Maheshwari, 2010	India	120	CT	8.7 ± 5.1	Consistent with low anteversion
Konar, 2016	India	180	CT	7.9 ± 6.2	−8 to 22
Debnath, 2016	India	100	Osteometric	20.1	Higher than other Indian
Patro, 2019	India	25	CT vs. XR vs. clinical	9.5 ± 5.3 (CT)	XR overestimated
Siwach, 2018	India	150	Radiograph + cadaver	Variable	Implants mismatch
Saikia, 2008	India	92	CT	20.4	Mongoloid > Caucasoid
Schmaranzer, 2019	Switzerland	52	CT (5 methods)	11–28	Method alters values
Scorcelletti, 2020	Global review	> 3,000	CT/MRI	15–20	Large variability
Deckey, 2022	USA	119	CT (MAKO)	6 (median)	45% retroversion
Lerch, 2023 (SCFE)	Switzerland / USA	49 hips	MRI	−1 ± 15	54% retroversion
Satpathy, 2015	USA	10 hips	Simulation	Retroversion ↑ stress	Supports impingement risk
Catani et al., 2018	Italy	362	Robotic CT (MAKO)	5.0 ± 9.6 (native FNV)	23% retroversion, moderate correlation FNV–SV, mean CV 28°
Current study, 2023	India	742	Robotic CT (MAKO)	−2.6 ± 11.9	53% retroversion

### Methodological considerations and measurement validity

Variability in reported femoral version across studies is strongly influenced by measurement technique and choice of distal femoral reference axis. Dorr et al., [23] reported a mean difference of 2.0° in femoral anteversion measurements when comparing transepicondylar axis (TEA) and posterior condylar axis (PCA) references, with excellent agreement (intraclass correlation coefficient = 0.994). Similarly, Castagnini et al., [24] evaluating 91 post-THA CT scans, identified a mean difference of 5.3° between TEA- and PCA-based measurements. Notably, they demonstrated superior interobserver and intraobserver reliability for TEA-referenced measurements compared with PCA, supporting the preferential use of TEA for assessing femoral stem anteversion. In a larger pre-THA cohort of 1,215 hips, Pierrepont et al., [6] reported a median femoral anteversion of 14.4° using PCA-based measurements, whereas the present cohort demonstrated a substantially lower mean femoral version of -2.6° (SD 11.9) and median value of -3° (IQR, -11 to 6). However, anteversion relative to the TEA was not evaluated in their study, limiting direct methodological comparison.

Schmaranzer et al. demonstrated that differences between commonly used CT measurement methods may exceed 15°–20°, particularly in hips with excessive torsion, underscoring the need for method consistency when interpreting femoral version values [22]. Similarly, Davis et al. reported a statistically significant but clinically small difference (mean 1.2°) between posterior condylar axis and transepicondylar axis referencing, supporting the validity of TEA-based measurement [13]. By using a uniform robotic CT protocol with automated landmark identification, the present study minimizes observer bias and intercenter variability, allowing for reliable population-level inference. This methodological approach may be particularly useful when evaluating population-specific anatomical variation.

### Sex-based differences

Consistent with prior literature, female patients demonstrated significantly greater anteversion than males, although absolute values remained lower than Western female cohorts [6, 25]. Sex-based dimorphism in femoral version has been repeatedly documented and is thought to reflect developmental and also biomechanical [13, 26]. Importantly, despite this relative increase, a substantial proportion of Indian female hips in the present cohort still demonstrated neutral or retroverted anatomy, reinforcing the inadequacy of applying fixed anteversion targets during THA [27]. 

### Etiology and age-related findings

No significant differences in native femoral version were observed across age groups or between avascular necrosis and primary osteoarthritis. This aligns with existing evidence suggesting that adult femoral torsion remains relatively stable after skeletal maturity, with minimal age-related remodeling [1, 28]. However, Parker et al., in their meta-analysis showed that variations in femoral versions may be associated with the development of hip osteoarthritis [29]. The cohort was heavily dominated by avascular necrosis (AVN), accounting for over 89% of cases, which represents a case-mix imbalance. This may limit the generalizability of the findings to primary osteoarthritis–dominant THA populations, where femoral morphology and degenerative changes may differ. The absence of an etiological association in the present study may be explained by the exclusion of hips with developmental dysplasia and other structural abnormalities; inclusion of such pathologies may be necessary in future investigations to better elucidate potential etiological influences.

### Clinical implications for total hip arthroplasty

The clinical implications of these findings are substantial. Application of Western-derived femoral anteversion targets in Indian patients may result in excessive combined anteversion, potentially increasing the risk of instability, particularly when using the posterior surgical approach [30, 31]. Femoral retroversion alters femoroacetabular mechanics and may predispose to impingement if not appropriately compensated by acetabular component positioning [2]. Marcovigi et al. demonstrated that low stem version markedly increases the risk of suboptimal combined version, even with robotic assistance [5]. Robotic CT-based planning may facilitate individualized assessment of femoral version and facilitates patient-specific optimization of combined anteversion [5–34]. Furthermore, Deckey et al. showed that native femoral retroversion is common in patients undergoing THA and is associated with abnormal spinopelvic mechanics, suggesting a compounded risk of impingement and instability if femoral anatomy is ignored [35]. In such scenarios, blindly forcing femoral stems into “textbook” anteversion may compromise canal fill, offset restoration, and biomechanical balance [36]. 

The high prevalence of femoral retroversion observed in this cohort has important implications for robotic-assisted THA planning. In patients with native femoral retroversion, reliance on fixed femoral anteversion targets may result in suboptimal combined anteversion and increased risk of impingement or instability. Additionally, attempts to forcibly increase femoral component anteversion should be avoided, as this may compromise canal fill and implant stability. Instead, a patient-specific combined anteversion strategy, integrating native femoral anatomy with optimized acetabular positioning, is recommended. The use of modular or version-adjustable stems may also be considered in select cases with extreme femoral retroversion.

The strengths of this study include its large sample size, multicenter design, and exclusive use of robotic CT-based measurement. Limitations include its retrospective nature, restriction to an Indian THA population, and lack of correlation with postoperative functional or survivorship outcomes. Another limitation is the exclusive inclusion of patients undergoing robotic-assisted THA. While this allowed standardized CT-based measurement using a uniform robotic planning protocol, it may limit external validity. Furthermore, femoral version values obtained using MAKO CT-based planning may not be directly interchangeable with values obtained using plain radiographs, conventional CT protocols, MRI, or manual landmark-based measurement techniques. Additionally, as this study did not include a comparative arm with conventional manual techniques, no conclusions can be drawn regarding the superiority of robotic-assisted planning. Given that 89.2% of the cohort comprised avascular necrosis, the findings may reflect a surgically selected population rather than the general Indian population. Although femoral torsion is believed to remain stable after skeletal maturity, the predominance of AVN may introduce selection bias, limiting generalizability [1]. Future studies should integrate spinopelvic classification, functional outcomes, instability rates and implant survivorship to determine whether population-specific femoral version norms translate into optimized implant positioning strategies and improved survivorship.

## Conclusion

This multicenter robotic CT-based analysis demonstrates that Indian patients undergoing total hip arthroplasty have substantially lower femoral neck anteversion than traditionally cited norms, with more than half exhibiting femoral retroversion. Significant sex-based differences were observed, while age and etiology had no meaningful influence. These findings underscore the importance of individualized, population-specific preoperative planning and caution against reliance on Western anteversion targets when performing THA in Indian patients.

## Data Availability

Available on reasonable request with corresponding author.

## Abbreviations

AVN: Avascular necrosis
CT: Computed tomography
FNA: Femoral neck anteversion
OA: Osteoarthritis
PCA: Posterior condylar axis
SD: Standard deviation
SPSS: Statistical Package for the Social Sciences
STROBE: Strengthening the Reporting of Observational Studies in Epidemiology
TEA: Transepicondylar axis
THA: Total hip arthroplasty
